# The Impact of Emergency Department Antibiotic Discordance on the Outcomes of Hospitalized Patients with Urinary Tract Infections: A Multi-Center Retrospective Cohort Study

**DOI:** 10.3390/life15040545

**Published:** 2025-03-26

**Authors:** Wing Yan Vivian Ng, Shou-Yen Chen, Hsien-Yi Chen, Chip-Jin Ng, Shi-Ying Gao, Chih-Huang Li

**Affiliations:** 1 Department of Emergency Medicine, Chang Gung Memorial Hospital, Linkou Medical Center, Taoyuan 33358, Taiwan; vivian422224@cgmh.org.tw (W.Y.V.N.); 8902007@cgmg.org.tw (S.-Y.C.); m7082@cgmh.org.tw (H.-Y.C.); nowl9@cgmh.org.tw (C.-J.N.); s78092359@cgmh.org.tw (S.-Y.G.); 2 College of Medicine, Chang Gung University, Taoyuan 33302, Taiwan; 3 Graduate Institute of Management, College of Management, Chang Gung University, Taoyuan 333, Taiwan

**Keywords:** urinary tract infection, treatment, concordance, outcome, length of hospital stay

## Abstract

Urinary tract infections (UTIs), particularly complicated ones, contribute significantly to emergency department (ED) visits and demand prompt treatment due to risks such as urosepsis. The effect of antibiotic concordance on patient outcomes with UTIs is unknown. We conducted a retrospective analysis from 2014 to 2023, focusing on those who received antibiotics for at least three days and had positive urine cultures. Patients were matched using propensity score matching to compare outcomes between those receiving concordant and discordant empirical antibiotics. We conducted propensity score matching to compare groups based on the initial empirical antibiotic’s pathogen sensitivity (concordant vs. discordant). Within our results, Escherichia coli emerged as the predominant pathogen (64.8%), but concordance varied widely among antibiotics, with Ertapenem showing the highest (91.3%) and Cefazolin showing the lowest rates (21.5%). There was no significant difference in mortality rates or ICU stays between the concordant and discordant groups. However, the hospital stay duration was notably shorter (11.5 ± 9.2 vs. 12.2 ± 9.4 days, *p* < 0.05) for patients receiving concordant treatment, indicating a faster recovery. Our findings suggest that, while choosing concordant antibiotics might not significantly affect mortality, it might be associated with a shorter recovery period during hospitalization. Choosing concordant antibiotics based on patient severity and punctually updated local sensitivity reports might minimize healthcare costs, and prevent ED overcrowding.

## 1. Introduction

Urinary tract infection (UTI) is a common condition diagnosed in the emergency department (ED); it accounts for 2 to 3 million ED visits every year in the United States [1,2]. UTIs are classified as either uncomplicated or complicated. In men, complicated UTI is characterized by fever, systemic illness, flank pain, costovertebral angle tenderness, or pelvic or perineal pain [3]. Urosepsis—sepsis originating from the urogenital tract—can cause multiple organ failure and hemodynamic instability if it progresses to septic shock [4]. The overall rate of mortality among patients with urosepsis is 30% [5,6].

*Escherichia coli* is the most common pathogen responsible for UTI and urosepsis (50%), followed by *Proteus* sp. (15%), *Enterobacter* sp. (15%), and *Klebsiella* sp. (15%) [5]. A definitive diagnosis of urosepsis requires positive urine and blood culture results. However, ED physicians should prescribe antibiotics immediately upon a preliminary diagnosis of UTI. The Sequential Organ Failure Assessment and Quick Sequential Organ Failure Assessment scores are widely used in the ED for their high specificity in diagnosing sepsis and their ability to guide prompt antibiotic treatment for patients with suspected urosepsis.

Although selecting antibiotics based on urine culture results is the most accurate approach for UTI treatment, it is often impractical because of the length of time taken to obtain culture results. Thus, empiric antibiotics are prescribed based on local susceptibility patterns and the patient’s previous urine culture results [7]. However, the growing prevalence of antibiotic resistance makes it difficult for ED physicians to select appropriate empiric antibiotics. Extended-spectrum beta-lactamase-producing Enterobacteriaceae are key bacteria that exhibit resistance to antibiotics such as penicillin, cephalosporins, and aztreonam [8]. Enterobacteriaceae resistant to fluoroquinolone and trimethoprim/sulfamethoxazole are responsible for >10% of all UTI cases in the United States [9]. The Infectious Diseases Society of America recommends avoiding antibiotics if the local resistance rate for the drug class exceeds 10% [9,10]. Risk factors for drug resistance include comorbidities, UTI episodes in the previous year, previous antibiotic exposure, indwelling catheters, and prolonged hospitalization [11,12].

The increasing prevalence of drug resistance often leads to the selection of discordant antibiotics in the ED [13]. Discordant antibiotic treatment also increases the risk of urinary infection-related hospital admission (UHA) for community-onset lower urinary tract infection [14]. However, it is still unknown how antibiotic discordant affects the outcomes of hospitalized UTI patients. In this study, we compared clinical outcomes between patients receiving concordant antibiotics and those receiving discordant antibiotics in the ED.

## 2. Materials and Methods

### 2.1. Study Design and Data Source

This retrospective study was conducted at Chang Gung Memorial Hospital (CGMH), one of the largest hospital networks in Taiwan. CGMH has branches in Linkou, Kaohsiung, Keelung, Taipei, and Chiayi. The Linkou branch, a tertiary medical center with a 3600-bed capacity, handles approximately 15,000 monthly ED visits.

The study protocol was approved by the Institutional Review Board of the Chang Gung Medical Foundation (permit number: 202301512B0, 13 October 2023). The requirement for informed consent was waived because of the retrospective nature of this study. Relevant data were obtained from the Chang Gung Research Database (CGRD), which contains deidentified electronic health records of patients from all branches of CGMH.

### 2.2. Study Population and Data Collection

Between 1 January 2014 and 31 July 2023, a total of 804,663 patients visited the ED of Linkou CGMH. Patients aged <18 years and those discharged directly from the ED were excluded from this study. The inclusion criteria were as follows: being prescribed antibiotics in the ED, using antibiotics for ≥3 consecutive days, and having UTI as one of the top three diagnoses at discharge.

The following clinicodemographic data were collected from the patients: age; sex; vital signs at presentation; comorbidities; complete blood count and differential count; and blood urea nitrogen, creatinine, total bilirubin, alanine transaminase, C-reactive, lactic acid, and procalcitonin levels. In addition, information on the antibiotics administered and the clinical outcomes (e.g., mortality rate and length of intensive care unit [ICU] stay) was collected.

### 2.3. Sample Collection and Antimicrobial Susceptibility Test

Urine samples were aseptically collected using a Foley catheter, or clean midstream urine was collected after appropriate sterilization.

An antimicrobial susceptibility test (AST) was performed using the Kirby–Bauer disk diffusion method and the Mueller–Hinton medium [15]. Bacterial sensitivity was tested against the following antibiotics: ampicillin, amoxicillin/clavulanic acid, ampicillin/sulbactam, piperacillin/tazobactam, cephazolin, cefuroxime, cefotaxime, ceftriaxone, ceftazidime, ciprofloxacin, levofloxacin, ertapenem, meropenem, imipenem, amikacin, gentamicin, and trimethoprim/sulfamethoxazole. Patients without AST results were excluded from this study (n = 6555). The final cohort comprised 33,606 patients.

### 2.4. Trends in Antibiotic Prescription and Resistance

Antibiotics commonly administered in the ED were grouped by their pharmacological properties. The initial antibiotics prescribed by ED physicians to the included patients (n = 33,606) were identified. These antibiotics were categorized and sorted by year for trend analysis.

The ten most common pathogens responsible for causing UTI in our patients were identified. The corresponding AST results were then obtained, and the resistance rate for each antibiotic was calculated. The data were organized as mentioned in the previous paragraph and subjected to trend analysis.

### 2.5. Statistical Analysis

Covariates including age, sex, initial vital signs, and comorbidities were used to calculate propensity scores in the multivariable logistic regression model. Each patient in the concordant group was matched with a counterpart in the discordant group. Categorical data are presented in terms of frequency and percentage values, whereas continuous data are presented in terms of mean ± standard deviation or median (Q1–Q3) values. Between-group differences were analyzed using Student’s t-test or the Wilcoxon signed-rank test for continuous variables and the chi-squared test or Fisher’s exact test for categorical variables. To minimize bias due to interpersonal differences, propensity score matching (1:1) was performed prior to between-group comparisons. Statistical significance was set at *p* < 0.05. Data were analyzed using Statistical Analysis System (SAS) (version 9.4; SAS Institute, Cary, NC, USA).

## 3. Results

### 3.1. Clinicodemographic Characteristics of the Patients

**Figure 1:**
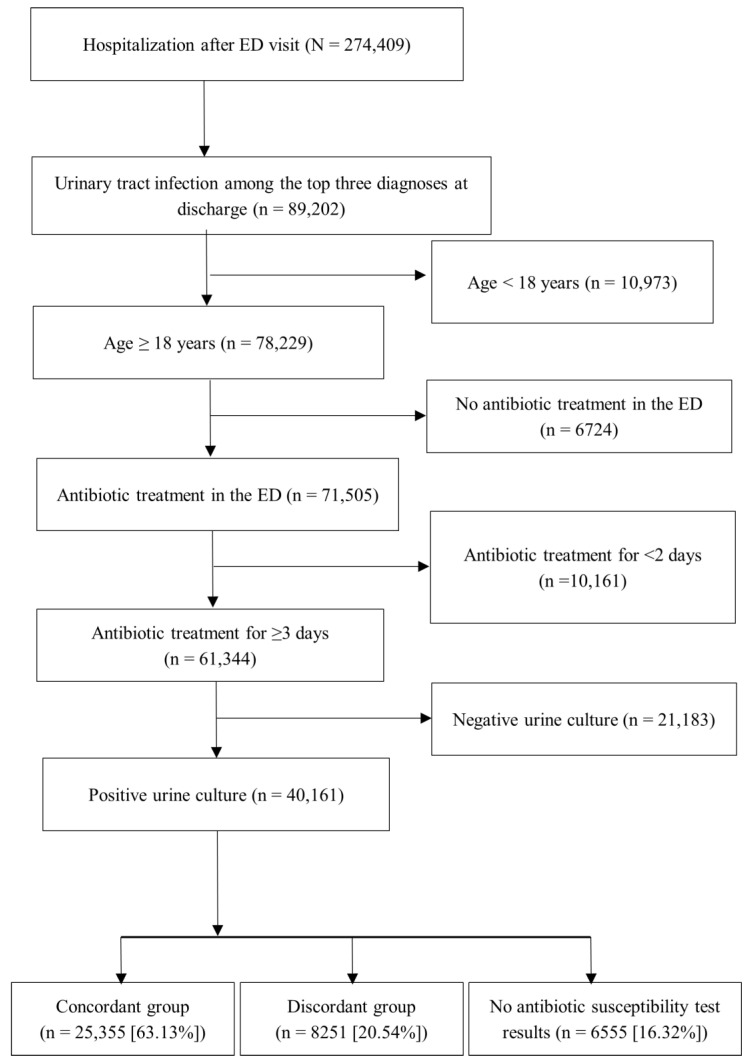
Flowchart depicting patient selection and group allocation. ED: emergency department.

Figure 1 presents a flowchart depicting patient selection and group allocation. During the study period, 78,229 patients were hospitalized after visiting the ED and received UTI as one of the top three diagnoses at discharge. Among them, 71,505 received antibiotics in the ED. We excluded patients who used antibiotics for <3 days or had negative urine culture results. A total of 40,161 patients had positive urine culture results. Among them, 6555 patients did not have AST results. Of the remaining patients, 25,355 carried pathogens susceptible to the first antibiotic prescribed in the ED (concordant group), whereas 8251 carried pathogens resistant to the prescribed drug (discordant group).

The patients’ mean age was 73.6 years. Of the patients, 68.6% were women (Table 1). The concordant group was younger than the discordant group. Furthermore, the proportion of female patients was higher in the concordant group than in the discordant group (5.7 ± 3.5; *p* < 0.0001). Initial vital signs were similar between the two groups. White blood cell counts, C-reactive protein levels, and procalcitonin levels were higher in the concordant group than in the discordant group. No significant between-group differences were observed in sepsis or septic shock prevalence. After propensity score matching, each group comprised 8251 patients. The matched groups did not differ significantly in age, sex, initial vital signs, comorbidities, laboratory findings, or sepsis or septic shock prevalence.

### 3.2. Results of Microbiological Assays

Table 2 presents the results of urine culture. The top 10 microorganisms identified in the urine samples were as follows: *E. coli* (64.8%), *Klebsiella pneumoniae* (9.8%), *Proteus mirabilis* (4.0%), *Citrobacter* spp. (3.1%), *Enterococcus faecalis* (3.0%), *Pseudomonas aeruginosa* (2.5%), *Enterobacter cloacae* complex (1.5%), *Morganella morganii* (1.2%), and vancomycin-resistant enterococci (0.9%). Patients carrying *M. morganii* (76.5%), *P. aeruginosa* (73.7%), and *P. mirabilis* (67.9%) had the highest likelihood of receiving concordant antibiotics. Conversely, patients carrying Vancomycin-resistant enterococci (42.4%), *Enterobacter* spp. (44.8%), and *E. coli* (47.4%) had the lowest likelihood of receiving concordant antibiotics. In patients with sepsis or septic shock, the risk of infection caused by pathogens other than *E. coli* increased from 33% to 45% during the study period.

### 3.3. Selection of Antibiotics

Table 3 presents information on the selection of antibiotics on the basis of concordance and disease severity. The 10 most prescribed antibiotics were as follows: ceftriaxone (40.4%), cefazolin (12.6%), cefuroxime (11.0%), cefoperazone/sulbactam (7.2%), ertapenem (6.8%), levofloxacin (6.7%), piperacillin/tazobactam (4.9%), ceftazidime (2.4%), ciprofloxacin (2.2%), and flomoxef (1.3%). Ertapenem (91.3%) exhibited the highest rate of concordance, followed by cefoperazone/sulbactam (77.8%) and piperacillin/tazobactam (66.3%). Cefazolin (21.5%) exhibited the lowest rate of concordance, followed by levofloxacin (34.7%) and cefuroxime (42.5%). We further analyzed the patterns of antibiotic selection for patients with sepsis or septic shock and those without it. Broad-spectrum antibiotics were prescribed more frequently to patients with sepsis or septic shock than to those without it. Cefoperazone/sulbactam, piperacillin/tazobactam, and cefepime were prescribed significantly more frequently than other antibiotics.

### 3.4. Clinical Outcomes After Treatment

Table 4 presents the outcomes of antibiotic treatment. No significant difference was observed between the concordant and discordant groups in the rate of in-hospital mortality (primary outcome; 3.9% vs. 4.2%; *p* = 0.437). The following outcomes (secondary outcomes) were similar between the two groups: rate of 30-day mortality (6.2% vs. 6.7%; *p* = 0.227), rate of ICU admission (2.4% vs. 2.1%; *p* = 0.17), length of ICU stay (11.4 vs. 11 days; *p* = 0.713), duration of inotropic agent use (4.5 vs. 3.6 days; *p* = 0.053), rate of intubation (1.4% vs. 1.6%; *p* = 0.177), and rate of hemodialysis upon admission (1.2% vs. 1.2%; *p* = 0.973). However, the length of hospital stay was significantly shorter in the concordant group than in the discordant group (11.49 ± 9.19 vs. 12.18 ± 9.44 days; *p* < 0.0001).

## 4. Discussion

UTI remains a prominent cause of hospitalization, particularly in older patients [16]. Approximately 30% of all patients hospitalized after visiting our ED had UTI as one of the top three diagnoses at discharge. The early diagnosis and management of UTI and subsequent sepsis or septic shock are crucial for preventing in-hospital mortality [17,18]. Because of differences in anatomical structure, women are more likely than men to have a UTI [19]. Approximately 65% of our patients had positive urine culture results; this proportion aligns with the literature. Negative urine culture results may be attributable to inadequate sterilization, mild infection with low bacterial load, or diagnoses made solely on the basis of clinical symptoms. The present study included patients with positive urine culture results, for whom AST results were available. At CGMH, bacterial sensitivity to antibiotics is determined by clinical pathologists and infectious disease specialists. Notably, the AST panel for *E. coli* comprises 12 antibiotics; however, a prescribed antibiotic may not always be part of this panel.

The most commonly reported UTI pathogens are uropathogenic *E. coli*, *K. pneumoniae*, *P. mirabilis*, *E. faecalis*, and *P. aeruginosa* [2,14,20]. In our study, *Citrobacter* spp. was the fourth most common pathogen. We included patients who received parenteral antibiotics and were hospitalized, because those treated with only oral antibiotics usually carry pathogens with low drug resistance.

The National Healthcare Surveillance Network highlighted the prevalence of *Pseudomonas* spp. in cases of complicated UTI. During the study period, the prevalence of *E. coli* decreased from 67% to 54.9%; reductions were also noted in the prevalence of *K. pneumoniae*, *E. faecalis*, and *P. aeruginosa*. However, the prevalence of less common pathogens (not among the top 10 bacteria) increased from 7.4% to 11.6%. On the basis of our findings, we recommend physicians to consider broad-spectrum antibiotics for patients with sepsis or septic shock.

Amoxicillin/clavulanic acid is the most commonly prescribed antibiotic for hospitalized patients with UTI, followed by ciprofloxacin and ceftriaxone [21]. For critically ill patients, piperacillin/tazobactam is frequently prescribed. Critically ill patients often receive multidrug therapy, with metronidazole and gentamicin being the most common agents [22]. We found that first-to-third-generation cephalosporins were the most commonly prescribed antibiotics during the study period. Ceftriaxone was the most frequently prescribed antibiotic because it could be used daily and required no renal dose adjustment. Cefazolin and cefuroxime were typically prescribed to patients without sepsis or septic shock. For patients with urosepsis, cefoperazone/sulbactam and piperacillin/tazobactam were preferred for their broad-spectrum coverage and low resistance rates. We noted that physicians tailored antibiotic prescriptions to their patients’ clinical conditions.

According to a local drug-resistance report, <70% of all *E. coli* isolates were susceptible to ceftriaxone, cefazolin, and cefuroxime. The concordance rate for these three antibiotics was only 21.5%. Resistance to third-generation cephalosporins and emergence of extended-spectrum beta-lactamase-producing Enterobacteriaceae are common causes of antibiotic discordance [13]. Levofloxacin exhibited a low rate of concordance in our study. However, these findings do not entirely preclude the use of these antibiotics as initial treatments. They may still be suitable for patients with uncomplicated UTI, a stable hemodynamic status, or previous urine culture results indicating sensitivity. For patients exhibiting signs of sepsis or septic shock, we recommend antibiotics with a high rate of concordance—for example, cefoperazone/sulbactam, ertapenem, piperacillin/tazobactam, and ceftazidime. Continuous monitoring of the hemodynamic status and the prompt de-escalation of antibiotics are necessary during the treatment course.

Evidence suggests that antibiotic discordance is associated with an elevated rate of mortality [6,23]. However, in this study, only the length of hospital stay was significantly shorter in the concordant group than in the discordant group, with no between-group difference observed in the rate of in-hospital mortality, rate of 30-day mortality, rate of ICU admission, length of ICU stay, or other secondary outcomes. This finding may be attributable to the overall severity of illness. Most of our patients did not meet the sepsis criteria, and the mortality rate was relatively low (4.2%). The patients did not develop sepsis or septic shock despite antibiotic discordance. The patients might be easier to develop drug resistance in certain condition such as spinal cord injury or indwelling catheter. A broad-spectrum antibiotic is mandated to prevent discordance. Also, physicians tend to prescribe broad-spectrum antibiotics during the study period to decrease the possibility of discordance. To prevent clinical deterioration, concordant antibiotics were prescribed to most patients based on their urine culture results.

Notably, the length of hospital stay was 0.7 days shorter in the concordant group than in the discordant group, suggesting rapid recovery with concordant antibiotics. Given the low overall mortality, clinical improvement and shorter hospital stays become significant indicators for a physician. Patients receiving discordant antibiotics might recover slower until antibiotic adjustment after available culture result. It usually takes two to three days in our organization. Some external confounders, including the standard care protocol, hospital supportive system, and antibiotic stewardship, might affect the patient’s recovery time. The effect of the confounding factor is minimal because all the study sites belong to one hospital system. The length of hospital stay is a crucial parameter for hospital administration. A shorter length of hospital stay is associated with lower healthcare costs and less ED overcrowding.

Our study has several notable limitations. First of all, the patients who had negative findings in urine culture were excluded in our study, which may have potentially caused selection bias. Second, there were no results regarding clinical improvement during the therapeutic period. The clinical parameter changes related to antibiotic discordance are unknown. Third, the availability of antibiotics might affect the overall antibiotic selection result. Most common antibiotics are available throughout the study except cefoperazone/sulbactam, which only became the drug of choice after 2018. Fourth, we did not compare the differences between hospitals, local physician preferences, or drug resistance patterns that might affect patient outcomes. Lastly, the study is retrospective in nature. A randomized-control study is best to determine the effect of discordant antibiotic treatment.

## 5. Conclusions

UTI remains a common cause of hospitalization. Shifts in pathogen prevalence and rising resistance rates challenge traditional treatment approaches. This study provides insights into antimicrobial prescribing practices for UTI in Taiwan. Pathogens other than *E. coli* increased in patients with sepsis or septic shock. Although antibiotics such as ceftriaxone are frequently prescribed, their low concordance rates with local sensitivity patterns call for careful consideration, particularly for use in critically ill patients. Patients receiving concordant antibiotics have relatively short hospital stays, which indicates rapid recovery and reduced healthcare burden. Therefore, antibiotics should be prescribed on the basis of pathogen sensitivity to optimize clinical outcomes, minimize healthcare costs, and prevent ED overcrowding.

## Figures and Tables

**Table 1:** Baseline characteristics of the patients.

	Before Propensity Score Matching			After Propensity Score Matching		
	Concordant (n = 25,355)	Discordant (n = 8251)	*p* Value	Concordant (n = 8251)	Discordant (n = 8251)	*p* Value
Age	73.0 (16.1)	75.4 (14.4)	<0.001	75.6 (14.1)	75.4 (14.4)	0.479
Gender (male)	7799 (30.8)	2768 (33.6)	<0.001	2752 (33.4)	2768 (33.6)	0.805
Charlson Cormobidity Index (CCI)	5.01 (3.5)	5.65 (3.5)	<0.001	5.63 (3.4)	5.65 (3.5)	0.679
Vital Signs						
Systolic blood pressure (SBP)	135.9 (31.6)	136 (30.7)	0.820	136.4 (32.9)	136 (30.7)	0.470
Mean arterial pressure (MAP)	96.2 (26.3)	96.4 (23.3)	0.515	96.1 (25.7)	96.4 (23.3)	0.514
Respiratory rate	19.8 (3.5)	19.8 (3.2)	0.515	19.9 (3.7)	19.8 (3.2)	0.200
GCS	15 (10–15)	15 (10–15)	<0.001	15 (10–15)	15 (10–15)	0.415
Laboratory data						
WBC count	12.7 (6.2)	12.0 (6.0)	<0.001	12.6 (6.3)	12.0 (6.0)	<0.001
Platelet count	222.1 (99.6)	227.7 (102.4)	<0.001	220.12 (98.0)	227.73 (102.4)	<0.001
Creatinine	1.5 (1.4)	1.5 (1.5)	0.812	1.7 (1.5)	1.5 (1.5)	0.047
Total bilirubin	1.0 (1.2)	1.0 (1.1)	0.042	1 (1.1)	1.0 (1.1)	0.414
Lactic acid	22.9 (17.3)	21.4 (15.4)	0.001	23.4 (17.7)	21.4 (15.4)	0.001
CRP	93.3 (88.6)	79.1 (79.1)	<0.001	92.6 (88.8)	79.1 (79.1)	<0.001
PCT	14.6 (33.3)	5.4 (15.7)	<0.001	11.0 (24.1)	5.4 (15.7)	0.002
Initial severity at ED						
qSOFA ≥ 2	4546 (17.9)	1500 (18.2)	0.619	1625 (19.9)	1500 (18.4)	0.011
Sepsis	4465 (17.6)	1463 (17.7)	0.815	1596 (19.3)	1463 (17.7)	0.008
Septic shock	22 (0.1)	4 (0.1)	0.391	9 (0.1)	4 (0.1)	0.267
No sepsis	20,890 (82.4)	6788 (82.3)	0.815	6655 (80.7)	6788 (82.3)	0.008

**Table 2:** Microbiological findings for patients stratified by concordance and disease severity.

Pathogens	n	%	Concordant n (%)	No Sepsis n (%)	Sepsis and Septic Shock n (%)
*Escherichia coli*	10,689	64.8	5067 (47.4)	9011 (67)	1678 (54.9)
*Klebsiella pneumoniae*	1623	9.8	891 (54.9)	1296 (9.6)	327 (10.7)
*Proteus mirabilis*	664	4.0	431 (64.9)	489 (3.6)	175 (5.7)
*Citrobacter* spp.	506	3.1	326 (64.4)	381 (2.8)	125(4.1)
*Enterococcus faecalis*	496	3.0	297 (59.9)	374 (2.8)	122 (4)
*Pseudomonas aeruginosa*	411	2.5	303 (73.7)	277 (2.1)	134 (4.4)
*Enterobacter cloacae* complex	241	1.5	108 (44.8)	206 (1.5)	35 (1.1)
B-*Streptococcus* Gr.B	202	1.2	101 (50.0)	150 (1.1)	16 (0.5)
*Morganella morganii*	166	1.0	127 (76.5)	153 (1.1)	49 (1.6)
*Enterococcus faecium* (VRE)	151	0.9	64 (42.4)	108 (0.8)	43 (1.4)
Others	1353	64.8	536 (39.6)	998 (7.4)	355 (11.6)

**Table 3:** Selection of antibiotics on the basis of concordance and disease severity.

Antibiotics	n	%	Concordant n (%)	No Sepsis n (%)	Sepsis and Septic Shock n (%)
Ceftriaxone	6670	40.4	3247 (48.7)	5643 (42)	1027 (33.6)
Cefazolin	2072	12.6	445 (21.5)	1911 (14.2)	161 (5.3)
Cefuroxime	1811	11.0	769 (42.5)	1617 (12)	194 (6.3)
Cefoperazone/sulbactam	1194	7.2	929 (77.8)	765 (5.7)	429 (14)
Ertapenem	1116	6.8	1019 (91.3)	920 (6.8)	196 (6.4)
Levofloxacin	1104	6.7	383 (34.7)	859 (6.4)	245 (8)
Piperacillin/tazobactam	815	4.9	540 (66.3)	463 (3.4)	352 (11.5)
Ceftazidime	388	2.4	253 (65.2)	273 (2)	115 (3.8)
Ciprofloxacin	359	2.2	163 (45.4)	314 (2.3)	45 (1.5)
Flomoxef	207	1.3	124 (59.9)	172 (1.3)	35 (1.1)
Others	766	40.4	379 (49.5)	506 (3.9)	260 (8.5)

**Table 4:** Treatment outcomes.

Matching	Total	Concordant (n = 8251)	Discordant (n = 8251)	*p* Value
In-hospital mortality rate	693 (4.2)	357 (4.3)	336 (4.1)	0.437
30-day mortality rate	1059 (6.4)	510 (6.2)	549 (6.7)	0.227
ICU admission rate	367 (2.2)	197 (2.4)	170 (2.1)	0.170
Length of ICU stay	11.23 ± 9.9	11.4 ± 10.0	11.0 ± 9.8	0.713
Length of hospital stay	11.84 ± 9.3	11.5 ± 9.2	12.2 ± 9.4	<0.001
Days using inotropic agents	4.11 ± 5.9	4.5 ± 6.5	3.6 ± 5.1	0.053
Intubation during admission	246 (1.5)	112 (1.4)	134 (1.6)	0.177
Hemodialysis during admission	196 (1.2)	97 (1.2)	99 (1.2)	0.943

## Data Availability

The datasets used and analyzed during the current study are available from the corresponding author on reasonable request.
